# Genome Sequence of Bovine Coronavirus Variants from the Nasal Virome of Irish Beef Suckler and Pre-weaned Dairy Calves Clinically Diagnosed with Bovine Respiratory Disease

**DOI:** 10.1128/mra.00821-22

**Published:** 2022-10-19

**Authors:** Kerrie Ní Dhufaigh, Matthew McCabe, Paul Cormican, Inmaculada Cuevas-Gomez, Mark McGee, Tara McDaneld, Bernadette Earley

**Affiliations:** a Teagasc, Animal & Grassland Research and Innovation Centre (AGRIC), Grange, Dunsany, Co. Meath, Ireland; b USDA, ARS, U.S. Meat Animal Research Center, Clay Center, Nebraska, USA; DOE Joint Genome Institute

## Abstract

We report 24 bovine coronavirus (BCoV) genome sequences from Ireland. BCoV was sequenced directly from nasal swabs that had been collected during a bovine respiratory disease (BRD) outbreak among recently purchased beef suckler and pre-weaned dairy calves.

## ANNOUNCEMENT

Bovine coronavirus (BCoV) is responsible for neonatal calf diarrhea, winter dysentery in adult cattle, and respiratory disease complex in cattle farms worldwide (1–3). BCoV (genus *Betacoronavirus* and family *Coronaviridae*) has a single-stranded positive-sense RNA genome of approximately 31 kb (4). We assembled BCoV genomes from sequences obtained directly from nasal swabs taken from 22 beef suckler weanling calves and 2 pre-weaned dairy calves in November 2019 (5) and March 2020 (6), respectively, after purchase from different marts and transport to the same research farm in the east of Ireland.

The study was approved by the Teagasc Animal Ethics Committee (TAEC-221-2019). Prior to nucleic acid extraction, 1× phosphate-buffered saline (PBS) was added to each nasal swab, which was then vortexed, and 300 μL of the eluate was removed and subjected to bead beating and then nuclease (RNase and DNase) treatment. A negative (blank swab with PBS) and a positive control (mixture of bovine respiratory syncytial virus [BRSV], bovine herpesvirus 1 [BoHV-1], and bovine parainfluenza virus 3 [BPI-3] bovine fetal lung cell culture isolates) was included alongside each batch of 10 sample extractions. Nucleic acids were extracted using the QIAamp MinElute virus spin kit (Qiagen, Manchester, UK) with carrier RNA substituted with 5 mg/mL linear acrylamide (Thermo Fisher). Double-stranded cDNA was synthesized using the Maxima H Minus ds-cDNA kit (Thermo Fisher) with random hexamers and purified using the GeneJet PCR purification kit (Thermo Fisher). Illumina libraries were generated using the VAHTS Universal Plus DNA library prep kit, and 150-bp paired-end sequencing was conducted on an Illumina NovaSeq.

Sequences were assembled with SPAdes (version 3.15) (7) and annotated using Prokka (version 1.14.6) (8). Adaptor sequences were removed, and reads were trimmed to remove low-quality bases using fastp (version 0.19.6). A total of 443,396,148 read pairs were generated. An average nucleotide identity of 99.78% was estimated using MEGA 11 between all 24 genome assemblies, which ranged in length from 30,879 to 31,216 bp. The BLAST nr/nt analysis (9) indicated all 24 Irish BCoV genomes were most similar to BCoV whole-genome assemblies from France (Table 1). All 24 Irish BCoV sequences shared 99% sequence coverage with their respective top BLAST nr/nt result, with the exception of two sequences, B0-128 and BB-78, which shared 100% sequence coverage.

**TABLE 1:** BCoV genome information relating to animal ID, GC content, genome size, GenBank accession nos., BLASTn results, and nucleotide identity (%)

Animal ID	GC%	Genome size (no. of nucleotides)	GenBank accession no.	SRA accession no.	Most similar BLASTn result (GenBank accession no.)	% identity of most similar BLASTn search
SeqDB-260	37	31,034	ON792964	SRR21722844	Bovine coronavirus isolate ICSA16-LBA (MG757140) or bovine coronavirus isolate ICSA-pool-EN (MG757141)	Both 99.03
SeqDB-205	37	30,957	ON792963	SRR21722940	Bovine coronavirus isolate ICSA-pool-EN (MG757141)	99.03
SeqBB-78	37	30,924	ON792958	SRR21530068	Bovine coronavirus isolate ICSA16-LBA (MG757140) or bovine coronavirus isolate ICSA-pool-EN (MG757141)	Both 99.13
SeqBB-129	37	31,005	ON792962	SRR21529975	Bovine coronavirus isolate ICSA-pool-EN (MG757141)	99.14
SeqBB-115	37	31,122	ON792961	SRR21529924	Bovine coronavirus isolate ICSA16-LBA (MG757140) or bovine coronavirus isolate ICSA-pool-EN (MG757141)	Both 99.13
SeqBB-110	37	31,122	ON792960	SRR21529957	Bovine coronavirus isolate ICSA16-LBA (MG757140) or bovine coronavirus isolate ICSA-pool-EN (MG757141)	Both 99.14
SeqBB-108	37	31,035	ON792959	SRR21530064	Bovine coronavirus isolate ICSA-pool-EN (MG757141)	99.16
SeqB0-88	36.9	31,061	ON792953	SRR21530062	Bovine coronavirus isolate ICSA16-LBA (MG757140) or bovine coronavirus isolate ICSA-pool-EN (MG757141)	Both 99.12
SeqB0-81	37	31,124	ON792952	SRR21530098	Bovine coronavirus isolate ICSA-pool-EN (MG757141)	99.14
SeqB0-79	37	31,060	ON792951	SRR21530099	Bovine coronavirus isolate ICSA16-LBA (MG757140) or bovine coronavirus isolate ICSA-pool-EN (MG757141)	Both 99.11
SeqB0-78	37	31,017	ON792950	SRR21530100	Bovine coronavirus isolate ICSA-pool-EN (MG757141)	99.14
SeqB0-74	37	31,078	ON792949	SRR21530102	Bovine coronavirus isolate ICSA16-LBA (MG757140) or bovine coronavirus isolate ICSA-pool-EN (MG757141)	Both 99.13
SeqB0-72	37	31,110	ON792948	SRR21530103	Bovine coronavirus isolate ICSA16-LBA (MG757140) or bovine coronavirus isolate ICSA-pool-EN (MG757141)	Both 99.15
SeqB0-70	37	31,061	ON792947	SRR21530105	Bovine coronavirus isolate ICSA16-LBA (MG757140) or bovine coronavirus isolate ICSA-pool-EN (MG757141)	99.09
SeqB0-66	37	31,079	ON792946	SRR21530106	Bovine coronavirus isolate ICSA-pool-EN (MG757141)	99.20
SeqB0-62	37	31,216	ON792945	SRR21530109	Bovine coronavirus isolate ICSA16-LBA (MG757140) or bovine coronavirus isolate ICSA-pool-EN (MG757141)	Both 99.13
SeqB0-59	37	30,968	ON792944	SRR21530111	Bovine coronavirus isolate ICSA16-EN (MG757139)	98.99
SeqB0-56	37	31,032	ON792943	SRR21530113	Bovine coronavirus isolate ICSA-pool-EN (MG757141)	99.12
SeqB0-54	37	31,042	ON792942	SRR21530115	Bovine coronavirus isolate ICSA-pool-EN (MG757141)	99.17
SeqB0-52	37	31,143	ON792941	SRR21530117	Bovine coronavirus isolate ICSA16-LBA (MG757140) or bovine coronavirus isolate ICSA-pool-EN (MG757141)	Both 99.12
SeqB0-128	37	30,879	ON792957	SRR21529922	Bovine coronavirus isolate ICSA-pool-EN (MG757141)	99.12
SeqB0-122	37	31,051	ON792956	SRR21529926	Bovine coronavirus isolate ICSA16-EN (MG757139)	99.02
SeqB0-116	37	31,102	ON792955	SRR21529928	Bovine coronavirus isolate ICSA16-LBA (MG757140) or bovine coronavirus isolate ICSA-pool-EN (MG757141)	Both 99.10
SeqB0-110	37	31,052	ON792954	SRR21529932	Bovine coronavirus isolate ICSA16-LBA (MG757140) or bovine coronavirus isolate ICSA-pool-EN (MG757141)	Both 99.14

The 1a, rep, 2a, HE, S, E, M, and N genes were identified from the Prokka analysis (8). The amino acid (aa) sequence of the hemagglutinin-esterase (HE) gene (GenBank accession number AAA66393) from the BCoV reference strain Mebus was aligned by MUSCLE in MEGA 11 (10, 11) to the 24 BCoV HE aa sequences. Relative to the reference strain, all 10 amino acid changes in the HE gene (L5P, N49T, S158P, F181L, N214S, S237A, E239Q, V362I, S367P, and L392I) were common to all 24 Irish BCoV genome assemblies. The N214S change is of interest, as it is in the receptor binding site (aa positions 211 to 214) of HE.

### Data availability.

The complete genome sequences of the BCoV/IRE/GR/2022 variants have been deposited in GenBank under the accession numbers ON792941 to ON792964.
